# The use of biomedicine, complementary and alternative medicine, and ethnomedicine for the treatment of epilepsy among people of South Asian origin in the UK

**DOI:** 10.1186/1472-6882-8-7

**Published:** 2008-03-20

**Authors:** Penny J Rhodes, Neil Small, Hanif Ismail, John P Wright

**Affiliations:** 1 Institute for Health Research, Temple Bank House, Bradford Teaching Hospitals NHS Trust, Bradford Royal Infirmary, Duckworth Lane, Bradford BD9 6RJ, UK; 2 School of Health Studies, University of Bradford, 25 Trinity Road, Bradford BD5 0BB, UK

## Abstract

**Background:**

Studies have shown that a significant proportion of people with epilepsy use complementary and alternative medicine (CAM). CAM use is known to vary between different ethnic groups and cultural contexts; however, little attention has been devoted to inter-ethnic differences within the UK population. We studied the use of biomedicine, complementary and alternative medicine, and ethnomedicine in a sample of people with epilepsy of South Asian origin living in the north of England.

**Methods:**

Interviews were conducted with 30 people of South Asian origin and 16 carers drawn from a sampling frame of patients over 18 years old with epilepsy, compiled from epilepsy registers and hospital databases. All interviews were tape-recorded, translated if required and transcribed. A framework approach was adopted to analyse the data.

**Results:**

All those interviewed were taking conventional anti-epileptic drugs. Most had also sought help from traditional South Asian practitioners, but only two people had tried conventional CAM. Decisions to consult a traditional healer were taken by families rather than by individuals with epilepsy. Those who made the decision to consult a traditional healer were usually older family members and their motivations and perceptions of safety and efficacy often differed from those of the recipients of the treatment. No-one had discussed the use of traditional therapies with their doctor. The patterns observed in the UK mirrored those reported among people with epilepsy in India and Pakistan.

**Conclusion:**

The health care-seeking behaviour of study participants, although mainly confined within the ethnomedicine sector, shared much in common with that of people who use global CAM. The appeal of traditional therapies lay in their religious and moral legitimacy within the South Asian community, especially to the older generation who were disproportionately influential in the determination of treatment choices. As a second generation made up of people of Pakistani origin born in the UK reach the age when they are the influential decision makers in their families, resort to traditional therapies may decline. People had long experience of navigating plural systems of health care and avoided potential conflict by maintaining strict separation between different sectors. Health care practitioners need to approach these issues with sensitivity and to regard traditional healers as potential allies, rather than competitors or quacks.

## Background

The United Kingdom (UK), in common with other western societies, is characterised by a plural health care system, comprising the dominant biomedical sector, with services delivered through the National Health Service (NHS) and by private providers, and a growing sector of so-called complementary and alternative therapies (CAM) [1] delivered by an array of mainly private providers [2], although there is increasing collaboration and interpenetration between the two sectors [3]. The most widely used CAM therapies, although often originating in specific cultural contexts, now have widespread global reach. In the context of Bradford, a city in the north of England in which the study was undertaken, we can add a third sector, that of traditional South Asian therapists, operating in parallel to but separately from the other two and without the global reach of biomedicine or global CAM [4]. In this paper, the phrase 'complementary and alternative medicines' is used to denominate those therapies originating outside the biomedical paradigm which have a widespread constituency reaching beyond the countries and ethnic groups in which they originated. The term 'ethnomedicine' is reserved for those traditional practices specific to particular ethnic groups, in this case South Asian groups.

CAM is now used widely in many western populations, especially for the maintenance of general health and the treatment of long term and medically intractable conditions [2]. Epilepsy might be considered a prime candidate for the use of CAM, as it is a chronic condition for which there is no medical cure, which may be medically intractable, or for which conventional medication (in the form of anti-convulsive drugs) may offer only partial control [5-7]. Studies have shown that, in western countries, a significant proportion of people with epilepsy use CAM specifically for epilepsy, usually in conjunction with rather than in place of conventional anti-convulsive therapy [8-12] and often without telling their doctors [12]. The most common CAM therapies used in the treatment of epilepsy include: mind-body therapies, such as reiki and yoga; biologic based therapies, such as herbal remedies, dietary supplements and homeopathy; manipulative-based therapies, such as chiropractic [8]. The web sites of the National Society for Epilepsy (UK) [13] and Epilepsy Action (UK) [14] devote whole sections to CAM, including: relaxation therapies, aromatherapy, acupuncture, reflexology, biofeedback, homeopathy, herbal medicine, Ayurveda, nutritional supplements and diet. CAM use is known to vary between different cultural contexts: for example, people with epilepsy in the United States of America are more likely to turn to prayer and other forms of religious healing than their UK counterparts [12,15]. However, little attention has been devoted to inter-ethnic differences within the UK population.

Among the South Asian sample studied, people turned to a combination of biomedicine and ethnomedicine but not to conventional CAM. In this article, we explore some of the reasons why. The Bradford study explored the use of ethnomedicine in a western, urban context, whereas previous studies of the use of ethnomedicine in relation to epilepsy have tended to be of traditional, non-western societies [16-20]. The article reports on the health care-seeking strategies of people from a migrant population and compares them with the profile of conventional CAM use in western societies and with the patterns prevalent in their homelands. The findings are likely to be relevant to other towns and cities worldwide where there are significant concentrations of South Asian settlement, as well as to migrant groups more generally.

### Study site and population

Bradford is the fifth largest metropolitan district in the UK and the eighth most socio-economically deprived health community [21,22]. Twenty per cent of the population of 380,000 people are of South Asian origin – from Pakistan, India and Bangladesh – and this proportion is projected to rise to 28% by 2011. The city's South Asian population is predominantly Muslim, of Pakistani origin and disproportionately represented in the most socio-economically deprived inner city wards. The 2001 census identified 14.4% of Bradford District's population as Pakistani, 2.7% Indian and 1.1% Bangladeshi. The South Asian community began to grow in Bradford as people moved from Pakistan in the late 1950's and 1960's to work in the textile mills and there has been continuing in-migration, often organised around marriage. There is now a growing cohort of older people but also a cohort of second and third generation people of South Asian origin, making for a complex and now long established community. Links with the Indian sub-continent remain close, with family and village ties sustained through in-migration and regular trips "home" by individuals or whole families.

The city has a community-based epilepsy service with a patient list of around 3000 [23]. Epilepsy clinics, in easily accessible local health centres, provide fast track consultations with newly diagnosed patients as well as monitoring patients who have had epilepsy for a period of time. The epilepsy service has been running for nine years and is headed by a full time consultant neurologist with a staff of four part-time specialist general practitioners (GPs) as well as a specialist epilepsy nurse and other support staff.

In parallel, and largely invisible to the NHS service, is a well-established network of community-based, traditional South Asian approaches to health care (see Table 1 for an over-view), which extends to other towns and cities where there are significant concentrations of people of South Asian origin. This network has close links with Pakistan and the Indian sub-continent. Healers who are resident in the UK are often allied to healers in India and Pakistan. Celebrated faith healers (*gurus *for Sikhs and Hindus, *pirs *for Muslims) and herbal practitioners (*hakims*) may be invited to the UK and tour the country providing religious healing. Traditional therapists are consulted and traditional remedies accessed when UK residents are visiting Pakistan. Consultations may be made without the recipient being present, either someone seeks help on their behalf or remedies can be obtained via correspondence: *tawiz *(amulets), for example, can be sent in the post.

**Table 1:** Features of the folk system

Religious healing involves individuals or groups praying or reciting religious texts to seek cure. Individuals may drink holy water, fast or undertake pilgrimages to seek forgiveness of sins and alleviation of illness
Faith healers (*gurus *– for Sikhs and Hindus, *pirs *– for Muslims) may be consulted for a number of illnesses/problems perceived to have a spiritual element (including epilepsy and mental illness). Some Muslims refer to such healers as *pirs*. The *pir*'s special spiritual power, acquired through birthright (lineage) or a lifetime of devotional acts, allows him to communicate directly with God and thus act as a mediator between God and the people.
*Pirs *offer a number of treatments. Amulets (*tawiz*), containing verses from the Koran are usually worn around the neck or on the arm, act as a defence against evil spirits or the evil eye (*buri nazaar*). Other types of amulets are dipped in water and then the blessed water is drunk. When the problem is thought to be spiritual, the *pir *may diagnose possession by evil spirits (*jinns*) which must be exorcised. However, only specialist *pirs *have the specific knowledge to perform exorcisms. While most *pirs *do not levy a charge for their services, they expect some type of payment in kind, often cash or jewellery. Others ask for donations to a mosque or other places in India and Pakistan that may be in need of charity.
There is a widespread belief amongst Muslims that *jinns *are spiritual beings – created from smokeless fire rather than the spirit of dead people – that live on earth in a world parallel to mankind. *Jinns *have the ability to possess and take over the minds and bodies of other creatures, including humans, and to behave in either a good or evil manner. *Jinns *possess people for different reasons. Most of the time possession occurs because the *jinn *is simply malicious and wicked.
The word *hakim *is derived from *hikmat*, the traditional system of medicine practised mainly among Muslim countries in South Asia. The practice, which involves use of a variety of herbs and minerals, has its basis in Greek medicine and Hippocrates' theory of the four humours (blood, phlegm, black and yellow bile). Humoural imbalance is thought to be the cause of ill health and it is the *hakim's *role to help regain the body's original state through a combination of medication (*dawaa*) and abstinence (*parhez*). In Pakistan, *hakims *often come from a well-established lineage of healers and are generally trained through a process of apprenticeship, although there are also a number of well-established institutions which offer training.

### Project management

In the initial stages of the study an Epilepsy Project Steering Group was established to provide general guidance to the project. Members included representatives from the funding organisation (Epilepsy Action), the hospital and community epilepsy service, the National Health Service Equality Strategy Unit, the local voluntary/charity sector (including South Asian community organisations), as well as the Director of Equality and Diversity at Bradford NHS Hospitals Trust and academic staff working in the fields of ethnicity and health care. The group met every six months to discuss sampling strategies, the topic guides used to gather data, and preliminary findings from the study. A Research Advisory Group, formed by research professionals, was established to discuss ongoing issues directly related to the management of the project and met on a monthly basis. In addition, a Patient Advisory Group of four people with epilepsy from the South Asian community, who were not themselves participants in the study, met with researchers to discuss the design of topic guides, interviewing strategies, preliminary findings and emerging data. However, recruitment to this group proved very difficult and it only met on three occasions. Members were paid expenses and taken out to a meal. It was not possible to make financial payments as these might have jeopardised their social security entitlements.

## Methods

### Obtaining the sample

After obtaining approval from the Local Research Ethics Committee, a sampling frame was drawn up using data from the city's Epilepsy Register of all patients referred to the community epilepsy service or seen by the hospital epilepsy team, with the following inclusion criteria: over 18 years old; resident in Bradford; of South Asian origin; diagnosed with epilepsy and receiving care from the hospital, epilepsy service or GP; no identified and recorded learning disability. The exclusion of potential participants on the grounds of learning disability was made on the advice of the project advisory group who thought that it presented too many confounding variables.

One hundred and thirty-nine people who met the inclusion criteria were identified using the computer programme, *Naam Pehchan*. This programme contains a dictionary of South Asian names that it attempts to match against the complete name, or name stem, in order to provide a list of South Asians, together with a language and religion marker for each person. Nam Pehchan has been assessed as having a sensitivity of 96% against names from Yorkshire, the county in which Bradford is located [24]. Different sources of information (Epilepsy Register, paper files from the Epilepsy Service and data from the Patient Administration System – PAS) were combined to collect information about each person's gender, GP, medication, address, telephone number and, where available, fluency in English.

The South Asian population is heterogeneous in terms of ethnicity, religions and language. Religious grouping provides a robust framework for reflecting some of this diversity and, to a large extent, can be correlated with socio-economic position [25]. Nazroo's work [25] demonstrates that, to a marked extent, the differentials relate to systematic differences in socio-economic position between Muslims (relatively poor) and Hindus and Sikhs (relatively better-off). In addition, other studies have shown that both ethnicity and religion provide an important framework for understanding South Asians' health behaviours and beliefs [26,27]. We therefore chose to divide our sample into Muslims, Sikhs and Hindus.

Given their low numbers, all Sikh and Hindu patients were included in the sample. The Muslims were grouped into gender and age bands (18–25, 26–35, 36–45, 46–55, 56–65, >65), and a quota sample drawn by selecting randomly from the age/gender bands and sending invitations to join the study accordingly. The European Standard population age bands were used, based on previous epidemiological research [23]. Patients were invited by letter, accompanied by an information sheet, (both in English and known or assumed first language) and asked to indicate on a return slip whether or not they wished to take part. Non-responders were further contacted by letter or telephone. Those who did not reply or who declined did not differ in terms of age banding or gender from the thirty who accepted the invitation to be interviewed. Tabulated breakdown of age and gender for responders and non-responders has been reported previously [28]. Reasons people gave for declining included: a belief that the research would be of no direct benefit to them personally, and not feeling comfortable talking about their epilepsy, especially as they feared that it might upset them and bring on a seizure. On a few occasions, relatives, acting as 'gatekeepers', did not allow the researcher to establish contact with the person with epilepsy and some potential participants contacted turned out to have learning difficulties (not recorded in the Register), and were excluded from the sample on the advice of the Epilepsy Project Steering Group. Table 2 provides a summary of the sampling and recruitment process.

**Table 2:** Process of recruiting people with epilepsy

**Identification of sampling frame**		
Identification of South Asian epilepsy patients from Bradford Epilepsy Register using computer program *Naam Pehchan *(n = 139)		

**Identification of sample**		

Inclusion of all Sikh and Hindu patients, random selection of Muslims from age and gender bands (n = 60)		

**Recruitment: Stage 1**		

Letters sent to patients (n = 60) (30 Muslims, 17 Hindus, 13 Hindus)		
Negative response (n = 3) (1 Muslim, 2 Hindus)	Positive response (n = 6) (5 Muslims, 1 Sikh)	No response (n = 51) (24 Muslims, 16 Sikhs, 11 Hindus)

**Recruitment: Stage 2**		

Telephone call or reminder letter (n = 51)		
Negative response (n = 8) (7 Muslims, 1 Hindu)	Positive response (n = 24) (15 Muslims, 5 Sikhs, 4 Hindus)	No response (n = 19) (2 Muslims, 11 Sikhs, 6 Hindus)

### Characteristics of the sample

The sample consisted predominantly of Muslims of Pakistani origin, reflecting the demographic profile of the local South Asian population, but also included Sikhs and Hindus. A total of 20 Muslims (10 male and 10 female), 6 Sikhs (2 male and 4 female) and 4 Hindus (3 male and 1 female) accepted the invitation. They ranged in age from 18 to 68 years, with 18 under 35 years of age. Five respondents classified their occupation as professional/managerial, six as skilled or unskilled manual, nine as housewives, eight as retired or unemployed (Table 3). The register data did not include information on socio-economic position or severity of epilepsy; however, the majority of participants were in lower socio-economic groups reflecting the socio-demographic composition of the city, and the eventual sample comprised individuals with a range of severity and adequacy of control through anti-epileptic drugs. Two patients reported other health problems (under-active thyroid and asthma) and seven people reported depression which they believed was directly linked to their epilepsy.

**Table 3:** Details of the 30 people with epilepsy included in the study

**Age Group**		**Type of Occupation**			**Household Structure**	
			**Male**	**Female**	**Lives with:**	
18–25	8	Professional/Managerial	3	2	Spouse only	3
26–35	10	Skilled manual	1		Spouse + children	11
36–45	5	Unskilled manual	3	3	Spouse + children + extended	8
46–55	4	Housewife		9	Spouse + extended	1
56–68	3	Retired	1	1	Extended only	1
		Student	2		Parents + siblings	2
		Unemployed	5		Parents only	2
					Siblings only	1
					Living alone	1

In recognition of the role of families in people's experience of epilepsy, the views of the person with epilepsy were supplemented, where possible, with those of another family member. Each person with epilepsy interviewed for the study was asked to nominate someone who cared for them in any capacity. Individual in-depth interviews were undertaken with fifteen carers who accepted the invitation to participate, of whom five were spouses, three parents, one a sibling and one a friend. Twelve people did not nominate a carer, either because they did not have a carer or did not want him or her to be approached, and three nominated carers (all husbands) declined because they felt uncomfortable about discussing their spouse's condition. Findings from the full study have been reported elsewhere [28-32].

### Interviews and analysis

Individual interviews were undertaken with the sample of 30 people with a diagnosis of epilepsy and 15 carers. The topic guides for persons with epilepsy and carers covered similar themes, including: lay knowledge of epilepsy; continuity and change; health and religious beliefs; use of alternative therapies; interaction with health professionals and level of satisfaction with service provision (Table 4). The topic guides were informed by a literature review and informal discussions with patients and health professionals, and were developed in collaboration with the research advisory group, which included representatives from local communities and the hospital and community epilepsy service. The basic content of the interviews was shaped by the topic guides to ensure that the same basic topics were raised in each interview, but additional topics were raised by the interviewees themselves. Most of the interviews were conducted by a male interviewer, one of the authors (HI), and five by a female researcher at the request of patients. Both interviewers were South Asian themselves, fluent in one or more South Asian languages, and experienced in the techniques of qualitative research. Interviews took place in participants' homes and lasted about one hour. Twelve of the interviews were conducted in Urdu or Punjabi at the request of participants, the rest in English, although interviews often combined more than one language. Nominated carers were interviewed separately, except on four occasions when joint interviews were conducted at the request of participants. All interviews were tape-recorded, translated by the interviewer if required, and transcribed. Difficulties in translation were discussed between the interviewers and with the rest of the research team.

**Table 4:** Summary of the Interview Topic Guide

**Topic heading**	**Interviewer prompts**
Nature of the study	Consent and confidentiality procedures: research aims
Personal information about informant	Demographics: household composition: family networks: length of time in UK: languages used.
Understandings about epilepsy	Diagnosis: beliefs about cause: impact on life: other health problems: knowledge of any other persons with epilepsy
Impact on lifestyle and relationships	Reactions of others – family/job/community: impact on what you can do: impact on mood: sources of support
Understandings about seizures	Frequency: severity: changes over time: triggers for seizures: reactions of others.
Experience of treatment provision	Language problems: medications: who would/do you go to for help: use of non-western therapies: satisfaction with care received.
Attitudes to the future	Beliefs about cure and control: ways to make things better.

The data were analysed using the framework approach [33]. This requires a coding frame to be devised based upon common themes and sub-themes found in the interviews. This is then applied to each transcript and relevant text is indexed whenever a particular theme appears. Coded segments of text are entered into a grid, specifying themes along one axis and (coded) respondent identities along the other. The range of responses can then be analysed by theme whilst retaining the integrity of individual interview profiles. Data from the interviews with carers were analysed separately. The initial coding was undertaken by the principal interviewer (HI) to ensure consistency and was reviewed by members of the research advisory group. Regular meetings of the research team and Research Advisory Group, where emerging themes were discussed, were held throughout the periods of fieldwork and analysis. Like Barbour, we took the view that 'the degree of concordance between researchers is not really important; what is ultimately of value is the content of disagreements and the insights that discussion can provide for refining coding frames. The greatest potential of multiple coding lies in its capacity to furnish alternative interpretations ...... Whether this is carried out by a conscientious lone researcher, by a team, or by involving independent experts is immaterial: what matters is that a systematic process is followed' [34]. Table 5 provides brief biographical details of those persons with epilepsy who are quoted in this paper. Names and precise details have been changed to preserve anonymity.

**Table 5:** Characteristics of those quoted*

**Name**	**Gender**	**Age**	**Religion**	**Occupation**	**Lives with**
Amjad	M	25	Muslim	Unskilled	Parents + other siblings and their families
Asifa	F	28	Muslim	Professional/clerical	Husband + children + mother
Banares	M	27	Muslim	Unemployed	Wife + parents + siblings
Bhupinder	F	46	Sikh	Professional/Managerial	Spouse + children + extended
Iqbal	M	35	Muslim	Unemployed	Spouse + children + extended
Khalida	F	56	Muslim	Professional	Husband + children
Mohammed	M	24	Muslim	Student	Grandparents and other members of extended family
Razia	F	31	Muslim	Housewife	Husband + children
Sachdev	M	19	Sikh	Unemployed	Parents + siblings
Saleem	M	32	Muslim	Unskilled manual	Spouse + children + extended
Santosh	M	42	Hindu	Unskilled manual	Spouse only
Sara	F	34	Muslim	Housewife	Husband +children
Shanaz	F	28	Muslim	Housewife	Husband + children + in laws
Shazad	M	23	Muslim	Unemployed	Parents + siblings
Yaqoob	M	39	Muslim	Unemployed	Wife + parents

* Names and precise details have been changed to preserve anonymity.

## Results

### Use of biomedicine

Not surprisingly, given their recruitment through medical records, all those interviewed were taking conventional anti-epileptic drugs for their epilepsy, although some (15) admitted to having stopped for brief periods in the past or to having altered the recommended dose.

I will have a fit if I'm still taking my tablet but, if I miss a tablet, I'm still going to have a fit. If I miss the tablet, I might not have a fit, you don't know. It's one of these things, it'll just come up from nowhere.

(Amjad, 25 year old Muslim man)

I was sick of taking tablets. I just thought I'd be my own doctor, basically. I thought, 'Well I have not had a fit for a very long time, I think it's gone away you know.' It's like, yes, it's finished, let's come off the tablets slowly.... I did have a quite bad fit.

(Sara, 34 year old Muslim woman)

The main reasons for stopping or altering the dose were: side-effects (such as headaches, loss of short-term memory, weight gain, impotence, stomach problems and general tiredness); fear of accumulated toxicity in the body; fear of harm to the unborn baby during pregnancy. Asifa was one of two women who had decided to stop taking medication for epilepsy during pregnancy.

No one knew about it. ... I was having cluster fits and the medication was put up and up and up. I was really scared that there would be some effect on the baby. I just thought, "I'm having the fits anyway, I'll have them but at least I won't be taking the tablets; the tablets won't be doing anything to the baby even if the fits are."

(Asifa, 28 year old Muslim woman)

In common with other young people with chronic illness, some participants, particularly if they had been diagnosed at a young age, went through a period in their adolescence when they stopped taking tablets [35-38].

When I was at that age, fifteen, I used to (take medication). It wasn't that I never understood properly, like if I had a headache you take a tablet, it goes away. I used to take the medication but I still used to have fits. In my head, I used to think 'What's the point of eating tablets?' so I stopped.

(Razia, 31 year old Muslim woman)

For many people, however, especially those who had experienced symptoms before coming to Britain, there had been a delay in diagnosis where help had initially been sought in the folk sector. As the brother of Iqbal (35 year old Muslim man) explained:

Magic, you know how it is in India, in Pakistan and Africa and everywhere. Yes, we thought it was the outside job and he went to see a lot of priests, different things, at first. He didn't know what it was.

### Use of complementary and alternative medicine

Unlike other studies which have revealed significant proportions of people with epilepsy using CAM, both for general health purposes and specifically for epilepsy [8-12], only two people mentioned a non-South Asian alternative. One had tried acupuncture, apparently without much success. Another, uniquely in our sample, had tried a number of complementary therapies, including psychotherapy, hypnotherapy, reiki and aromatherapy. Although his views about the benefits of using alternatives were mixed, he was opposed to trying any kind of remedy proposed by a traditional South Asian healer:

They (parents) have tried to (persuade me) but I just know I don't want to see any of these people (traditional healers). No, because it's just superstition... I'd rather do it my own way. I did use aromatherapy. That helped with sleeping and relaxing me and I tried reiki as well..... Reiki was through my brother and aromatherapy, his partner. She has books and things on it, so I just borrowed a book and read that.... I just tried it to see how it would help me..... I've tried some things like how to become stress-free and things but I don't feel it really works.

(Sachdev, 19 year old Sikh man)

### Use of traditional South Asian therapies

Despite high levels of compliance with western medication, the use of traditional South Asian therapies was widespread in our sample, although in all cases as a second-line rather than an alternative to anti-epileptic drugs. Given recruitment of the sample through medical records, we have no knowledge of the extent to which people with epilepsy may have been using traditional therapies without being in contact with medical services. Initially, participants were often reluctant to talk about the use of ethnomedicine, even with interviewers who were themselves of South Asian origin, and no-one reported having discussed it with their doctor.

Over half the sample (16 people) said that they had sought help from *hakims*, practising mainly within the Yunani tradition [39], or from other traditional South Asian healers. Fourteen people said that they had visited a faith healer. Some of these consultations were with *gurus *(Hindus and Sikhs) or *pirs *(Muslims), visiting from the Indian subcontinent, although some people consulted local healers on trips abroad when visiting extended family. Others consulted religious healers established in the UK, often affiliated to famous *pirs *from the subcontinent, on the recommendation of friends or family.

Decisions to seek alternative treatments were usually taken as a family rather than as an individual and people reported having been coerced or cajoled into accepting them, especially on visits to India or Pakistan where there was additional pressure from the wider family and community. One man, for example, remembered how:

My mum forced me, saying, "You have to go, you try it there. What are you going to lose if you go there?" and I had to accept those things.

(Yaqoob, 39 year old Muslim man)

Similarly, another recalled:

(I went to a faith healer) just to please them (my parents), really. They were saying that, not only my parents, it was like far off cousins or my uncles, they would say, "You should go. Someone went, he got better, and why don't you take your son?"

(Banares, 27 year old Muslim man)

Discrepancy between participants' reasons for accepting traditional treatments and families' motivations was commonly reported and was usually related to differences in beliefs about the causes of epilepsy. The older generation were reported to be more likely to believe in supernatural causes, such as the Evil Eye, *tawiz *(amulets, containing verses of the Holy Quran, intended to harm someone), or possession by mischievous demons or *jinn *[40,41]. As the husband of Asifa pointed out:

They (the elders) think it's something like an evil spirit, I would say that's the elders, no matter where they are.

Younger people were more likely to accept a medical explanation for their condition or to attribute it to stress, past trauma and "the will of God". Yaqoob, for example, said:

Most people think like that that they are djinns (demons) and want to give you tawiz (amulet containing religious verses) or something like that.... It's a disease, it's not ghost. It's a disease, it's not a ghost giving me trouble. If it's a ghost, it could kill me a long time ago.

(Yaqoob, 39 year old Muslim man)

Similarly, Sikh and Hindu respondents were unlikely to subscribe to the notion of punishment for sins committed in a past life. Bhupinder, for example, commented:

They do say that you have to repent in this life for sins that you've maybe committed in your previous life, but I don't know... I mean illness is something that's fated to happen to you in life. I think I was just fated to have it.

(Bhupinder, 46 year old Sikh woman)

Likewise, Sachdev, who was diagnosed at the age of 12, recalled:

I remember (my parents) having conversation with some guru of theirs and he was saying that in a previous life ...I'd killed a snake or something, that's why I'm epileptic... but I don't really believe in this sort of thing.

(Sachdev, 19 year old Sikh man)

Older relatives were likely to consider biomedicine inferior to traditional approaches with their strong religious foundation. The father of Shazad (23 year old Muslim man) explained:

In this country people usually go to the doctor first, because it's free and the hakim charges money. In Pakistan, however, the hakim is cheaper than the doctor.... I think the hakim's cure is better, the cure is from Allah and both are ways of accessing the cure.

Similarly, Saleem recounted a conversation with his father after visiting a doctor. Previously, he had visited a *pir*, visiting from Pakistan, who had diagnosed the problem as *saya *(literally meaning 'shadow' – the belief that some evil spirit or individual has cast his or her shadow on the patient [41]) and given him some verses to read in Farsi.

Me and Dad went to Dr X a few months later. He showed us a circuit board and he said these wires touch and connect. Afterwards, when we came back home, Dad said "Allah ne bandyi" (Pir was a person close to God and not everyone is blessed with these powers), you know, he said "Not everyone has that."

(Saleem, 32 year old Muslim man)

Amongst participants, the widespread use of traditional therapies was not necessarily or even primarily related to belief in their efficacy. Participants turned to alternatives for a number of reasons. Some were persuaded by family, friends or relatives; others were concerned about side-effects, especially the effects on their health of long-term use of anti-epilepsy drugs. Some, mirroring their approach to anti-epileptic drugs, had adopted an experimental tactic. One woman, for example, had tried both herbal remedies prescribed by hakims and religious remedies, but was sceptical of their benefit:

I also went to see the hakims. People that give prayers to read to get better, I went to see them too. I tried everything and sometimes I'd feel better, sometimes I wouldn't. But, I mean, they say it's kind of psychological as well. Well, you know, you take it (hakim's prescribed medication) and you don't have any fits for a week and then the next week you'll have three and you think, "What was the point of taking that, it hasn't made any difference?"

(Shanaz, 28 year old Muslim woman)

Similarly, Mohammed had stopped wearing *tawiz *because he felt they hadn't helped him.

I had a couple sent (from Pakistan) a couple of years ago... Got it from M (a pir in Pakistan)... But when I have these attacks, I just have these doubts, again... Then, after two or three years I was in that previous state when I used to keep on having fits again and again, I thought they weren't working so I just took them off, just stopped using them.

(Mohammed, 24 year old Muslim man)

However, a common motivating factor, regardless of age, religion or ethnicity, was the desire for a cure or reduction in seizures that conventional medication had failed to provide. All those who had sought additional treatment had experienced continued seizures, despite compliance with medication [42], and many gave a sense of desperation as their main motivation.

We've been asking all those sorts of people but nobody can come up with any answer. They've tried their best ... but nothing... I think it is desperation because, you know, you've been trying something for so long and it's not getting you anywhere... I don't care as long as it helps me. Anything, I was willing to do anything.

(Santosh, 42 year old Hindu man)

I've had people giving me tawiz, all sorts. I've been... I don't know, people say different things: "You should do this, you should do that." I've tried everything.

(Razia, (31 year old Muslim woman)

One person had even sought help from a healer of a different religion:

We must have tried nearly everything, anybody that said, you know, 'Try this for epilepsy.' It's like go an Islamic way going beyond it. It's like something you wouldn't do, you know. My family is quite religious and sometimes we actually went to this (Hindu healer). There's nothing wrong with that but, I suppose, you know, when you've got your child, you're helpless, you don't know what to do, you run to everyone, you don't care who you're going to or what religion they are.

(Sara, 34 year old Muslim woman)

In line with findings from studies conducted in India [40,43] and Pakistan [44], participants expressed considerable scepticism about the efficacy and authenticity of both the treatments and their practitioners but were willing to go along with them out of a sense of desperation or to please their families. However, some had refused to follow the healers' instructions. Khalida, for example, recounted an occasion when her mother had taken her to consult a *pir*.

It was in Pakistan. The pir, I don't know what he did and, after that, he said "Can you open your mouth?" and I said, "Why?" because he wanted to spit in my mouth. I said "No, I won't". My mother said, "You must obey" I said "I don't think so." Yeah, it's our people, they believe in these things. I don't believe.

(Khalida, 56 year old Muslim woman)

Others had simply refused to see them. Asifa, for example, had refused to see a religious healer even though her family had insisted:

Someone once suggested, I think it was in the family, you know, some peer sahib is really good, he will do some dua (recite prayers) or something... but I don't really believe in that stuff.

(Asifa, 28 year old Muslim woman)

Others refused to wear the *tawiz *(amulet) they had been given.

We've got this other one (pir) who comes to your house... That's what our lot believe in. He's all right, he gives tawiz and recites from the Koran, still our faith. I've never worn them (tawiz), don't believe in them.

(Amjad, 25 year old Muslim man)

Some questioned the authenticity of practitioners, believing many to be charlatans or quacks.

I mean, I'm sure there are many true people out there but they're difficult to find, aren't they? So, most of them are just quacks basically, so... I don't believe in that stuff.

(Asifa, 28 year old Muslim woman)

(My aunt) has been to these pirs, fakirs whatever you want to call them, one in Pakistan that helped her, she's been to see all of them. But half of them are conmen, you know that. They go round taking money off you.

(Amjad, 25 year old Muslim man)

Muslims queried the role of the *pir *as an intermediary with God [45,46]. One woman, for example, said:

I don't believe in pir. Okay, you respect them because maybe they know bit more than you. I am a pir as well, I'm God's human being, why won't he listen to me? Why should I go to another person to tell my problems? You pray to God. Why can't I pray myself?

(Khalida, 56 year old Muslim woman)

Others were ambivalent about the wearing of *tawiz *and other magico-religious practices that were perceived to run counter to Islamic orthodoxy [47] and were associated with the "superstitious" village culture of rural Pakistan. A young man explained:

(W)hatever God plans, he's the best of all plans and I can't, can't go to someone and say, 'Give me a tawiz and make me feel better.' If I'm going to get better, then God will make me better. (I went) just to please them (parents) really.

(Banares, 27 year old Muslim man)

## Discussion

### Summary of findings

All those interviewed were taking conventional anti-epileptic drugs for their epilepsy, although for some people, who had experienced symptoms before coming to Britain, there had been a delay in diagnosis where help had initially been sought in the folk sector. The use of ethnomedicine was widespread, although in all cases as a second-line rather than alternative to anti-epileptic drugs; only two people mentioned a non-South Asian therapy. No-one had discussed the use of traditional therapies or CAM with their doctor. The decision to seek traditional treatment was usually taken at the family rather than individual level, with younger people often having limited power to resist decisions taken on their behalf. Many were ambivalent about the efficacy and safety of some traditional therapies. Participants turned to traditional therapies for a variety of reasons; however, a common motivating factor was a desire for a cure or reduction in seizures – all those who had sought additional treatment had experienced continued seizures, despite compliance with medication.

### Limitations of the study

Epilepsy is presented as an example of a chronic condition, for which conventional biomedicine has limited efficacy and for which people are known to turn to CAM. Although health care-seeking strategies in response to epilepsy may not be representative of health care seeking strategies more generally, they are likely to be similar to those in response to other chronic, incurable conditions where conventional biomedicine has limited efficacy.

The sample was self-selecting in the sense that people chose whether or not to take part and it may therefore have been more likely that people who were compliant with medical regimens were included. In view of the relatively low reported prevalence of epilepsy in the city's South Asian population [23], the Epilepsy Register may under-represent the actual number of people of South Asian origin with epilepsy. Our sample may therefore have been unrepresentative of the overall population of people with epilepsy because it did not include people who did not access or were not known to NHS services. These possible biases, if anything, strengthen our finding that the use of ethnomedicine is prevalent within the South Asian population studied, since we would expect its use to be even more widespread among people who do not adhere to conventional medical regimens or who are managing their condition outside the NHS.

A further possible bias might have been introduced with the conduct of joint interviews or of individual interviews conducted in the presence of other family members. Some people may have felt inhibited or embarrassed discussing or expressing their opinions about certain issues in front of relatives. The use of ethnomedicine and, in particular, certain traditional beliefs and practices may be one of these issues. As we discovered, the use of ethnomedicine was often a source of disagreement, and sometimes open conflict, within families. Moreover, the sensitive nature of the topic, the association of certain beliefs and practices with an illiterate peasantry and their characterization as superstition and quackery among some orthodox Islamic circles [45-47] may have inhibited some people from discussing them with a middle class, university-educated interviewer who had been born in the UK. Again, any such potential bias would only add weight to the findings.

### Comparison with global CAM users

The health-seeking behaviour of the people in our sample in respect of non-biomedical treatments, although mainly confined within the traditional folk sector, shared much in common with the patterns of behaviour and motivations of people who use global CAM in the treatment of epilepsy. Like global CAM, traditional South Asian therapies were used as a supplement rather than alternative to conventional drug therapy [12,48]. Similarly, it was the failure of Western medicine to offer a cure or adequate improvement to their condition which prompted people to turn elsewhere for help. One reason for seeking additional therapy was dissatisfaction with conventional treatment – dislike of side-effects from conventional therapy, concerns about the harmful effects of long term drug use, and inadequate seizure control.

However, here the similarities seem to end. Except for one person, whose behaviour as a serial or multiple CAM user marked him out from the rest of the sample, the portrayal in the literature of people acting as autonomous individual consumers breaks down. Unlike the use of global CAM, which is often linked with the growth of individualism, identity politics and consumerism [49-54], selecting an appropriate treatment was rarely an individual's decision, especially for children and young people who were reliant on parents to make such decisions for them. The family and wider extended kin and community network played an important role in making and guiding choices [55] and, in this context, notions of individual empowerment, personal autonomy and control over health care decisions had little salience. Younger participants, in particular, often felt they had little active choice in decisions to seek traditional treatments.

In the context of the study, it was important to disaggregate the use of ethnomedicine from the decision to seek treatment. Those who made the decision to consult a traditional healer and the recipients of the treatment were not necessarily the same people and their respective motivations, perceptions of safety and efficacy were often very different.

Unlike mainstream users of CAM, participants' use of traditional therapies was not necessarily, or even primarily, related to belief in their efficacy; in fact, many were highly sceptical. Similarly, there was little evidence of the 'philosophical confluence' [56] thought to motivate conventional CAM use. Amongst recipients (if not their families), resort to traditional therapies was not necessarily underpinned by belief in their underlying philosophy and many of those who used (or were subjected to) traditional treatments explicitly rejected traditional theories of causation.

However, these differences become less sharply defined when we take into account the way in which and by whom decisions to use ethnomedicine were taken. To the parents of participants, the attraction of traditional therapies may well have lain in their compatibility with their own (as opposed to their children's) values, philosophy and beliefs regarding the nature and meaning of epilepsy. Many in the Pakistani community in Bradford emigrated from rural villages of the Mirpur region of Azad Jammu Kashmir and most first generation immigrants have an impoverished rural background where people were heavily reliant on traditional practitioners in the absence of accessible and affordable alternatives. In addition, unsatisfactory encounters with mainstream medical services in the UK, resulting largely from language and cultural barriers [29,57-61], may have encouraged people to turn to the folk sector with which they were more familiar.

### Comparison with patterns of CAM use in India and Pakistan

In many ways, the patterns observed amongst study participants in the UK mirrored those reported among people with epilepsy and other conditions in India and Pakistan. These included: the use of traditional remedies not necessarily linked with perceptions of greater efficacy or safety [4,43,56,62]; reported discrepancies between the views of patients and their families [4,43,56,62]; decisions about where to seek treatment taken at the family rather than individual level [41,43,55,56]; the influence on health care-seeking behaviour of relative accessibility and cost of different options [41,43]; the importance of lay referral networks [41,43].

In India and Pakistan, most people are managed by primary care resources and have little access to specialized investigations or personnel [43]. People's choices are heavily constrained, with much of the rural population having only limited access to public services [63]. Accessibility, cost and the reliability of supply of medicines have been shown to be important determinants of people's health seeking strategies [43,56] and indigenous approaches are often a first resort [41,43,56]. For those with mental illness or epilepsy, which is often popularly classed as a mental condition [41,56], faith healers may often be a preferred option [41].

In the UK, in our sample, this pattern was reversed, with traditional treatments used as a supplementary or second line resort. However, their use remained high. This may reflect the attraction of familiar institutions and traditions which have the additional reassurance of offering explanations for epilepsy from within people's own belief system (as opposed to medical uncertainty) and the prospect of a cure (as opposed to control) [30,56]. Resort to traditional therapies filled a space left vacant by mainstream medicine, specifically its (frequent) failure to provide adequate explanation, control or cure and the burden of side-effects resulting from long term use of anti-epileptic drugs. Other commentators have highlighted the holistic approach of traditional South Asian therapists [64,39] and, in this, South Asian therapists share much in common with the approach of other CAM practitioners.[65] However, this was not something to which participants in the study alluded.

Continued use of traditional therapies by migrants may be associated with nostalgia or with a desire to maintain a distinctive ethnic identity [66]. However, in our study, we found no evidence to support this thesis. In the plural health care systems of India and Pakistan, the simultaneous use of different treatment modalities is common. Participants and their families may simply have been continuing patterns of behaviour prevalent in their homelands [66].

It is likely that much of the appeal of traditional South Asian therapies, as opposed to mainstream CAM, lay in their familiarity and religious and moral legitimacy within the South Asian community, especially to those of the older generation who were disproportionately influential in the determination of treatment choices. However, the views about the legitimacy, value and likely efficacy of traditional therapies of those who decided to seek treatment within the folk sector (mainly first generation migrants) were often at variance with those of recipients of the treatment (mainly second generation), which were often ambivalent, sceptical and even hostile. As the second generation come to take the place of the first generation as decision-makers, patterns of health seeking behaviour are likely to change.

Traditional South Asian therapies have a long history of usage in the South Asian population studied, with practitioners chosen on the basis of reputation and personal recommendation. [39,41,43,66] In this, they share much in common with practitioners of other CAM therapies, for whom personal referrals and knowledge about alternatives obtained through the lay referral network have been found to be 'a powerful legitimizing influence' [50]. But, although the operation of lay referral networks was important in the use of traditional South Asian therapies, on the whole, participants did not appear to have had access to similar networks in respect of other CAM therapies, revealing, perhaps, the relative isolation of much of the Bradford South Asian community from the lay referral networks operating in other sections of society [66].

## Conclusion

### Implications for practice

Several arguments have been advanced as to why clinicians should be interested in CAM (and ethnomedicine). First, an increasing number of patients are now using CAM, both for general health purposes and specifically for the treatment of epilepsy: clinicians need to be aware that it may feature among the options being actively considered by patients. Second, with the growth of the Internet, unorthodox treatments will continue to garner publicity and their claims will continue to seem attractive to patients [11]. Third, it is argued that clinicians owe it to their patients to investigate the possibility of finding less toxic ways of improving quality of life in epilepsy [11] and, fourth, that interest in alternative treatments can offer new and innovative ways to study epilepsy, 'a group of conditions that we still know little about despite several decades of intensive worldwide study' [11]. Finally, and perhaps most importantly, some CAM treatments have been found to be harmful in their own right or in interaction with anti-convulsive drugs [67-71]: greater clinical awareness can prevent adverse interactions.

However, patients may not be forthcoming in telling their doctors about their use of CAM [12] and this reticence may be even more pronounced among people who use ethnomedicine [16]. In the Bradford study, patients rarely, if ever, discussed the use of ethnomedicine with medical practitioners. Such reticence has its roots in a history of colonialism and the promotion of western biomedicine, initially as the preserve of the white colonialists. There remains today a strong class dimension with biomedicine used primarily by the more affluent and educated urban population and traditional healers consulted by the less educated, rural poor. In many western societies, the medical establishment has begun to reach some form of accommodation with CAM practitioners (albeit reluctantly) and there has been increasing interpenetration of the two sectors [3]. However, the relationship between indigenous practitioners and doctors practising within the biomedical tradition in India and Pakistan is still characterised by mutual hostility and mistrust. Indigenous practices have come under increasingly negative scrutiny from the medical profession both in India and Pakistan and in the UK [56,67-71]. The National Society for Epilepsy, for example, now issues specific warnings against the use of Ayurvedic preparations [13]. Even where health professionals are themselves South Asian, class and educational differences and fear of judgemental attitudes may inhibit open communication. Where practitioners and their patients do not share a common language and cultural background, communication about such sensitive issues is even less likely to be forthcoming [72].

In common with other non-western groups, among the Bradford sample, the spheres of ethnomedicine and orthodox biomedicine were kept distinctly separate [16]. People had long experience of plural systems of health care and had learned how to make choices: they used the services of biomedicine where it was perceived to be efficacious [16,73] and were adept at moving between the different spheres without experiencing dissonance or contradiction [28,30]. Indeed, they avoided potential conflict by maintaining a strict separation. Health care practitioners need to learn about their patients' cultural backgrounds and to approach these issues with care and sensitivity, if they are to breach this barrier. They need to "learn how to ask" [74]. They need to regard traditional healers as potential allies, rather than competitors and quacks, and to learn how to work with them to explore ways in which different approaches can supplement and complement each other. In the context of globalised CAM, this momentum is gathering speed; in the context of South Asian ethnomedicine, the dialogue has yet to begin.

## Competing interests

The author(s) declare that they have no competing interests.

## Authors' contributions

PR contributed to the design and supervision of the study and wrote the substantive paper.

NS contributed to the design and supervision of the study and to drafts of the paper.

JW was the grant holder and responsible for management and supervision of the study.

HI undertook data collection and much of the writing of the original report.

All authors read and approved the final manuscript.

## Pre-publication history

The pre-publication history for this paper can be accessed here:


